# Effect of Storage Time After Surface Treatment of Zirconia on Surface Free Energy and Bond Strength of Composite Cement

**DOI:** 10.3290/j.jad.c_2562

**Published:** 2026-03-11

**Authors:** Mina Aker Sagen, Heidi Vanessa Holm, Freya Andersen, Per Vult von Steyern

**Affiliations:** a Mina Aker Sagen Researcher, Nordic Institute of Dental Materials, Sognsveien 70a, 0855 Oslo, Norway. Study design, conducting the study, analyzing the data, writing and reviewing the manuscript.; b Heidi Vanessa Holm Engineer, Nordic Institute of Dental Materials, Sognsveien 70a, 0855 Oslo, Norway. Study design, conducting the study, writing and reviewing the manuscript.; c; d Freya Andersen Engineer, Nordic Institute of Dental Materials, Sognsveien 70a, 0855 Oslo, Norway; Helly Hansen AS, Munkedamsveien 35, 0250 Oslo, Norway. Study design, conducting the study, writing and reviewing the manuscript.; e Per Vult von Steyern Professor, Nordic Institute of Dental Materials, Sognsveien 70a, 0855 Oslo, Norway; Faculty of Odontology, Malmö University, Sweden. Study design, analyzing the data, writing and reviewing the manuscript.

**Keywords:** Al2O3, etching, resin cement, shear bond, surface treatment, zirconia

## Abstract

**Purpose:**

Surface treatment of zirconia before cementation can be performed using different methods, e.g. airborne particle abrasion (APA) or various etching protocols. This study evaluated the effect of storage time between surface treatment and cementation on the surface free energy (SFE) of zirconia, the bond strength of composite cement, and failure mode.

**Materials and Methods:**

Rod-shaped zirconia specimens were fabricated and assigned to two surface treatment groups: APA (n = 80) and hot etching with potassium hydrogen difluoride (KHF_2_, n = 80). Each group was divided into four storage time subgroups: immediate, 24 h, 1 week, and 1 month. After storage, specimens were either analyzed for SFE (n = 10) or cemented for shear bond strength (SBS) testing and failure mode evaluation (n = 10).

**Results:**

Two-way analysis of variance (ANOVA) showed a significant effect of both surface treatment and storage time on SFE (P < 0.05), with KHF_2_-etched zirconia exhibiting the highest values across all time points. In both groups, SFE gradually decreased with increased storage. Surface treatment did not significantly affect SBS (P > 0.05). Storage time significantly influenced SBS (P < 0.05), specifically for KHF_2_-etched zirconia; post-hoc comparisons showed higher SBS after 1 week than at immediate testing (P < 0.05). Although adhesive failures to cement increased with longer storage time for KHF_2_-etched specimens, this trend was not significant (P > 0.05). For APA specimens, the highest incidence of adhesive failures (n = 9) to cement occurred after 24 h of storage.

**Conclusion:**

Given the significant decrease in SFE with prolonged storage and adhesive failures to zirconia tended to increase over time, minimizing the interval between surface treatment and cementation is recommended.

Zirconia was introduced as a dental restorative material in the 1990s. Owing to its favorable mechanical properties and gradually improved translucency, it has become one of the most widely used ceramic materials for indirect restorations.^7,19^ However, due to its composition and crystalline structure, achieving reliable adhesion between zirconia and composite cement remains challenging for predictable long-term bonding to tooth substance.^23^ Zirconia is characterized by densely packed polymorphic crystals (monoclinic, tetragonal, cubic)^5^ and lack of an amorphous phase, which distinguishes it from other ceramics for dental applications, like glass ceramics. Consequently, surface treatment of zirconia to enhance adhesion of composite cement has been comprehensively investigated over the last decades^17^ and various laboratory and chair-side methods are now available,^9,27,33^ all aiming to promote chemical bonding,^18^ increase the intaglio surface area, create mechanical interlocking of the cement^9,11,18,22,31^ and enhance surface free energy (SFE) for improved wettability.^35^ Most frequently used method is airborne particle abrasion (APA) with aluminum oxide particles (Al_2_O_3_).^10,17^ The abrasion parameters, such as particle size, pressure, duration, distance from the nozzle, and angulation to the surface, vary among studies.^11,13^ However, the most commonly used approach is particles in the range of 30–60 µm applied to the surface with a low pressure at a 75–90-degree angulation from a 10 mm distance for 5–10 s.^10^ Numerous studies^3,11,33,34^ have shown that APA, followed by the application of primers containing phosphate monomers (10-MDP), produces a strong and durable bond between composite cement and zirconia, showing that APA is an effective pretreatment method for restorations.

Another method that roughens and activates the bonding surface of zirconia is hot etching with potassium hydrogen difluoride (KHF_2_).^25,29^ Although documentation of this surface treatment’s effect on the bond strength of composite cement is limited, published studies report results comparable to those of APA.^1,29^ The melt of KHF_2_ creates surface irregularities similar to those created by hydrofluoric acid (HF) etching of glass ceramics, thereby promoting micromechanical interlocking with the cement.^29,30^ Additionally, etching increases SFE, which improves surface wettability.^1,25^ The anticipated formation of hydroxyl groups (OH) on the surface may also promote chemical bonds to primer and composite cement.^29,30^

Due to equipment availability and/or safety considerations, surface treatment is mainly performed in the dental laboratory before a restoration is sent to the dentist. Consequently, the interval between surface treatment and cementation may vary, and the restoration may be stored for several days before placement in the patient’s mouth. However, the effect of storage time between surface treatment and cementation on the bond strength of composite cement is scarcely documented in the literature. In a published study by Al-Akhali et al,^2^ APA zirconia was stored up to 72 h before SFE measurement and bond strength testing. The authors concluded that increasing the time between APA and cementation negatively affected both SFE and bond strength; therefore, zirconia should be cemented shortly after surface treatment. The conclusion was based on an increasing number of adhesive failures between zirconia and cement with longer storage, indicating surface contamination from the surroundings.

Given the limited evidence regarding the effect of storage time between surface treatment of zirconia (APA or KHF_2_ etching) and cementation, the present study hypothesized that prolonged storage time would reduce the SFE of zirconia, thereby decreasing bond strength and altering failure mode. Hence, the following null hypotheses were tested: (1) Storage time between surface treatment (APA or KHF_2_ etching) of zirconia and cementation has no effect on SFE, SBS, or failure mode, and (2) There is no difference between the two surface treatment methods (APA or KHF_2_ etching) with respect to SFE, SBS, or failure mode.

## MATERIALS AND METHODS

### Specimens

Circular-shaped zirconia cylinders (n = 160) were fabricated from pre-sintered discs (KATANATM Zirconia, Super Translucent Multi Layered, Kuraray Noritake Dental, Tokyo, Japan) using a CAD/CAM technique. The cylinders were designed using CatiaTM V5.21 (Dassault Systemès, France) and transferred to K5+, vhf (Ammerbuch, Germany) milling machine for milling under dry conditions.

The zirconia cylinders were sintered according to the manufacturer’s instructions (1550°C with a hold time of 2 h. Temperature increase/decrease of 10/–10°C/min respectively). Post-sintering dimensions of the cylinders were: d: 5 mm, h: 10 mm. The bonding surfaces of the cylinders were left in their original states to simulate the intaglio surface of a restoration.Post-production, the cylinders were stored dry.

Circular composite discs (n = 80, d: 7 mm, h: 4 mm) (Filtek One Bulk Fill Restorative, A3, 3M, St. Paul, MN, USA) were built in layers of 2 mm using a plastic mold. Each layer was light-cured for 60 s using a LED curing unit (2100 mW/cm^2^, Demi Ultra, Kerr, Orange, CA, USA). The composite discs were further embedded in acrylic resin (ClaroCit, Struers, Ballerup, Denmark) using mounting cups (SeriForm, Struers, Ballerup, Denmark). The bonding surface of composite discs was exposed, and mechanically ground using P600 SiC paper to obtain a uniform surface roughness. Post-production, discs were stored in distilled water at 37°C.

### Surface Treatment

The zirconia cylinders were divided into two surface treatment groups: (1) APA with 50 µm aluminum oxide particles (Al_2_O_3_, Korox, Bego, Bremen, Germany) (n = 80) using a pressure of 1 bar from a 10 mm distance for 15 s. The nozzle was held perpendicular to the surface and moved sideways for a uniform abrasion^18^; (2) hot etching with potassium hydrogen difluoride (KHF_2_, Honeywell, Charlotte, NC, USA) (n = 80) at 300°C for 10 min.^29^ After both surface treatment methods, cylinders were thoroughly steam cleaned and ultrasonically cleaned in distilled water for 15 min. Finally, they were air-dried using oil-free pressurized air.^29^

Cylinders in each surface treatment group were randomly divided into four different storage times, resulting in eight groups (n = 20) (Table 1). Specimens were stored dry in closed containers in a dark environment at room temperature.

**Table 1:** The eight test groups

Storage time	Airborne particle abrasion	KHF_2_ hot etch
Immediate	APA-im	KHF_2_-im
24 hours	APA-24 h	KHF_2_-24 h
1 week	APA-1 we	KHF_2_-1 we
1 month	APA-1 mo	KHF_2_-1 mo
im – immediate; 24 h – 24 hours; 1 we – 1 week; 1 mo – 1 month.		

After storage, cylinders in each group were randomly divided manually by one operator into SFE measurement (n = 10) or shear bond strength (SBS) testing and failure mode evaluation (n = 10).

### Surface Free Energy

SFE was measured based on the contact angle between two liquids of known surface tension and the treated zirconia surface. Cylinders were dripped with 2 µL of either deionized water or diiodomethane and the contact angle of the liquid was measured using the sessile drop method (Drop Shape Analyzer DSA30, Krüss, Hamburg, Germany) and calculated according to the Young-Laplace fit (ADVANCE 1.16.1 software, Krüss, Hamburg, Germany).^38^ For each cylinder and liquid, ten measurements were taken immediately after deposition of the drop, and the average was calculated. Testing was conducted at room temperature. Based on the contact angle of both liquids, the polar and dispersive components of the SFE were determined using the Owens, Wendt, Rabel, and Kaelble (OWRK) method,^26^ the sum of which makes up the total SFE of the cylinder surface.

The surface area of a single cylinder was not large enough for a drop of both measuring liquids. Therefore, within every test group, half the cylinders were dripped with deionized water and half with diiodomethane. Cylinders with differing testing liquids were randomly paired and the two contact angles were used to calculate one SFE value. Consequently, there were five SFE measurements for every test group of ten cylinders.

### Cementation

Specimens for bond strength testing were created by cementing the zirconia cylinders to the composite discs embedded in acrylic resin using a dual-cure composite cement (Panavia V5, Kuraray Noritake Dental, Tokyo, Japan). Prior to cementation, a ceramic primer (Clearfil Ceramic Primer Plus, Kuraray Noritake Dental, Tokyo, Japan) was applied to the zirconia surface and air-dried, whereas a tooth primer (Tooth Primer, Kuraray Noritake Dental, Tokyo, Japan) was applied to the composite disc for 20 s and air-dried, in accordance with the manufacturer’s instructions for use.^20^ A standardized seating load of 10 N was applied during cementation using a jig. Excess cement was removed using a micro-brush. Finally, the cement was horizontally light-cured using an LED curing unit (2100 mW/cm^2^, Demi Ultra, Kerr, Orange, CA, USA) for 20 s from four directions separated by 90 degrees.

### In Vitro Aging Protocol

After cementation, the test specimens were stored in distilled water at 37°C for 24 h and thereafter, thermally cycled 5000 cycles in 5°C and 55°C water baths with a holding time of 20 s for *in vitro* aging.

### Shear Bond Strength

For SBS testing, specimens were mounted in a universal mechanical test machine (zwickiLine Z5.0, ZwickRoell, Ulm, Germany) and applied shear force using a knife-edge-shaped cross-head at a speed of 1 mm/min. The horizontal distance between the cement space and the cross-head was less than 0.5 mm. Shear force (N) at breakage was registered and MPa calculated for each specimen using software (testXpert III-v1.3, ZwickRoell, Ulm, Germany) connected to the universal testing machine.

### Failure Mode

After SBS testing, both zirconia cylinders and composite discs were studied in a light microscope (NexiusZoom NZ.1903-S, Euromex, Arnhem, Holland) to determine failure mode: (1) adhesive between zirconia and cement; (2) adhesive between composite and cement; (3) cohesive in cement; (4) mixed failure; a combination of the above-mentioned failure modes (non of the failure modes comprises three-quarters or more of the surface). Selected zirconia cylinders from all test groups were studied in a scanning electron microscope (SEM, TM4000Plus, Hitachi, Tokyo, Japan) to confirm the failure mode registered in the light microscope.

### Statistical Analysis

The number of specimens in each group was selected based on previously published studies on surface treatment and bond strength with a similar approach.^2,29^ Statistical analysis was conducted using R-4.3.0 (R Core Team, 2023). The assumption of normality was evaluated using the Shapiro–Wilk test and found to be satisfied. For the bond strength data, a two-way ANOVA was conducted to assess the effects of surface treatment and storage time, and their interactions. When statistically significant effects were detected, post-hoc comparisons were conducted using Tukey’s HSD test. For the SFE data, an independent sample t-test was used to compare differences between surface treatments at each storage time. This test was selected because the SFE measurements for each surface treatment and storage condition were obtained from independent groups. A Fisher’s exact test was used to assess whether there was a significant relationship among the different failure mode groups. The significance level was set at α = 0.05.

## RESULTS

**Fig 1a and b:**
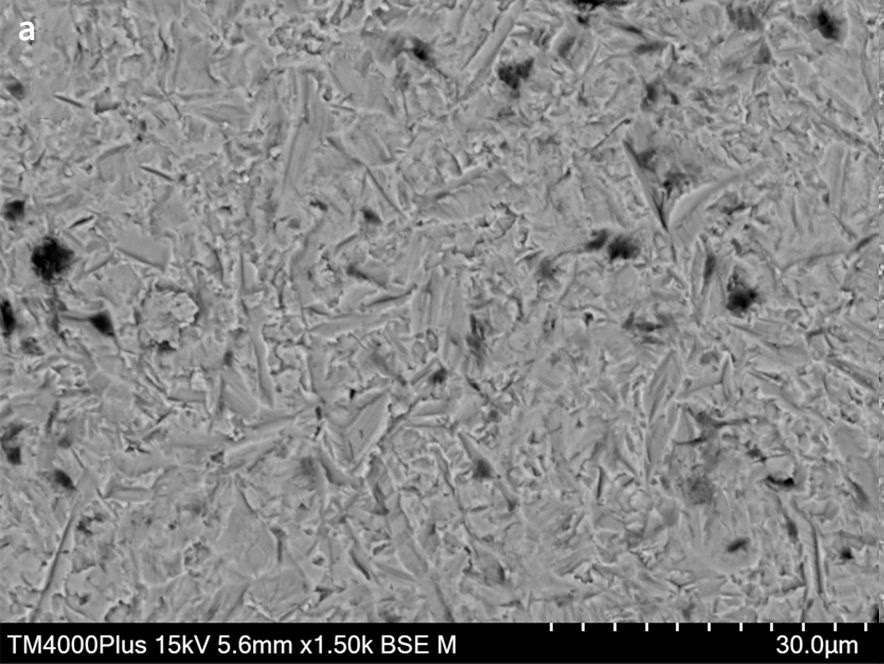

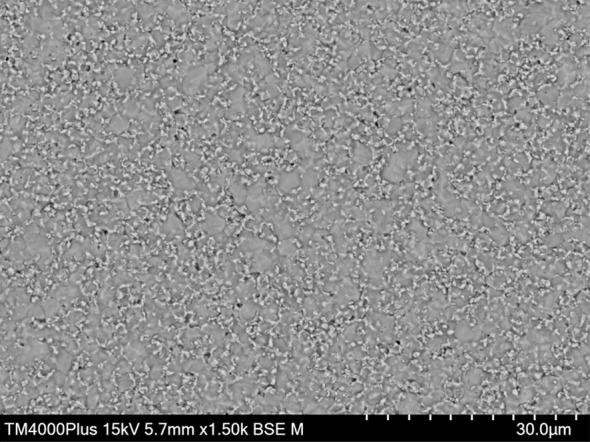
SEM images showing the zirconia surface structure after airborne particle abrasion (a) and KHF_2_ etching (b).

Surface structure after APA and KHF_2_ etching is shown in Figure 1.

### Surface Free Energy

**Fig 2:**
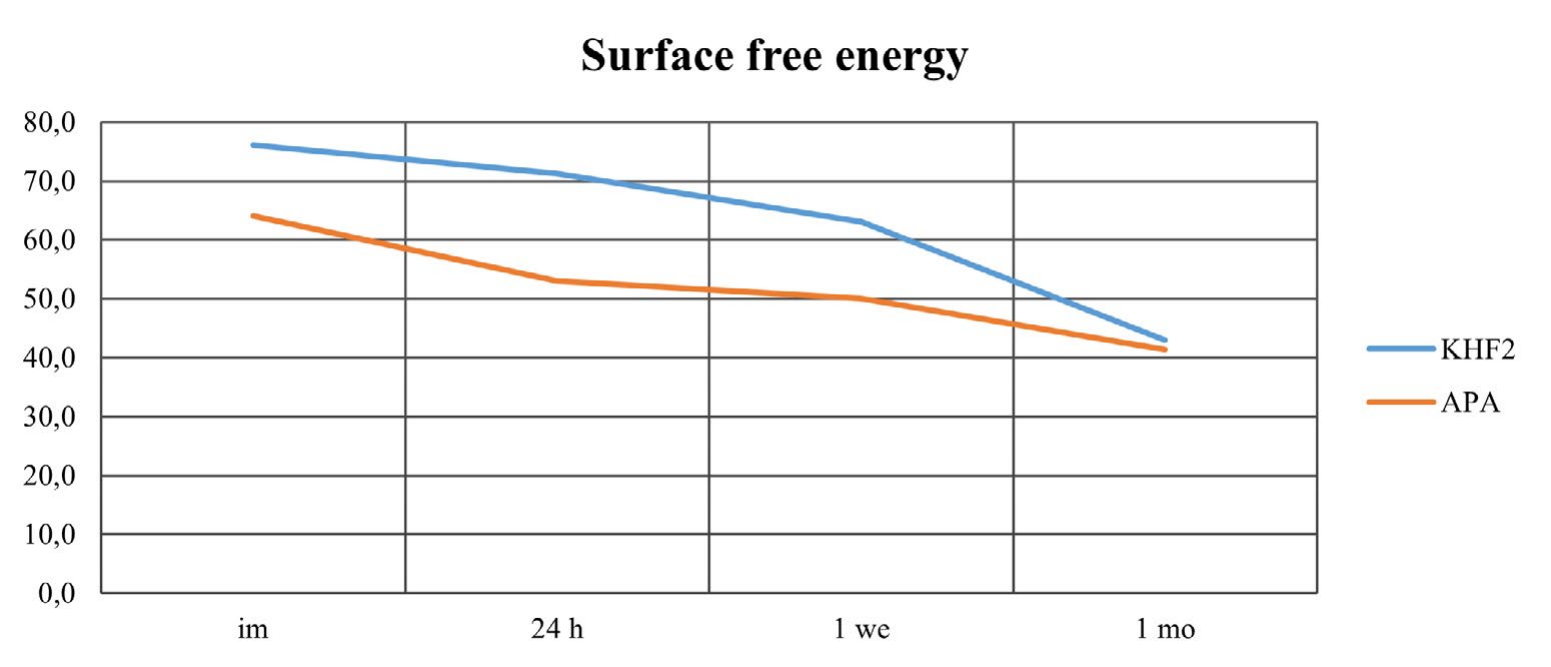
Mean SFE (mN/m) of KHF_2_-etched and airborne-particle-abraded zirconia (APA) specimens after four different storage times. im – immediate; 24 h – 24 hours; 1 we – 1 week; 1 mo – 1 month.

The highest SFE values were observed immediately after surface treatment (Fig 2). Two-way ANOVA showed that both surface treatment and storage time had a significant effect on SFE (P < 0.05). For both treatments, SFE decreased significantly with increasing storage time (P < 0.05), except for between the immediate and 24 h groups for KHF_2_-etched zirconia, and between 24 h and 1 week for APA zirconia (P > 0.05).

Independent t-test indicated that KHF_2_-etched zirconia generally exhibited higher SFE values than APA zirconia, with significant differences (P < 0.05) observed immediately, after 24 h and 1 week storage; however, the difference after 1 month was not significant (P > 0.05).

Regarding the polar components of SFE, a significant decrease (P < 0.05) was observed from immediate to 1 month storage for both surface treatment groups (Table 2).

**Table 2:** Mean SFE and (standard deviation) in mN/m with dispersive and polar parts in each group

Group	SFE	Disperse	% Disperse	Polar	% Polar
APA-im	64.1 (1.1)	41.6 (1.6)	65	22.5 (2.7)	35
APA-24 h	53.2 (4.7)	36.8 (2.0)	69	16.4 (5.1)	31
APA-1 we	50.2 (3.0)	39.1 (4.2)	78	11.1 (1.3)	22
APA-1 mo	41.7 (2.4)	35.9 (1.1)	86	5.9 (2.3)	14
KHF_2_-im	75.6 (2.9)	46.1 (3.4)	61	29.5 (3.6)	39
KHF_2_-24 h	70.6 (5.2)	43.7 (0.6)	62	26.8 (4.8)	38
KHF_2_-1 we	62.9 (4.6)	41.1 (2.0)	65	21.8 (4.8)	35
KHF_2_-1 mo	45.8 (5.9)	37.5 (1.0)	82	8.3 (6.3)	18
im – immediate; 24 h – 24 hours; 1 we – 1 week; 1 mo – 1 month.					

### Shear Bond Strength

**Fig 3:**
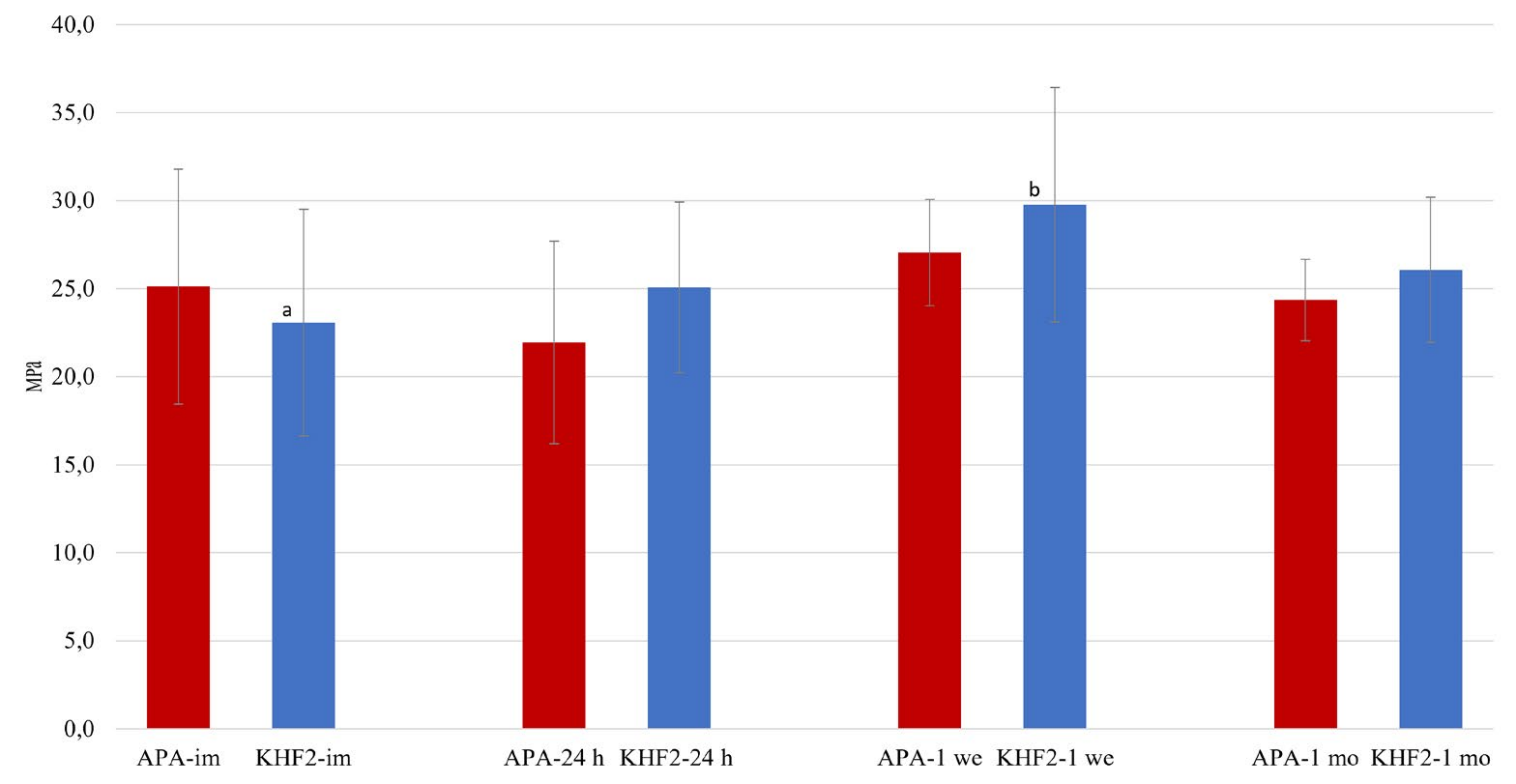
Mean shear bond strength and standard deviation in MPa for KHF_2_-etched and APA zirconia. Significant differences within surface treatment groups are marked with different letters. im – immediate; 24 h – 24 hours; 1 we – 1 week; 1 mo – 1 month.

Two-way ANOVA revealed that surface treatment method did not have a statistically significant effect on SBS (P > 0.05) (Fig. 3). In contrast, storage time significantly influenced SBS (P < 0.05), specifically for KHF_2_-etched zirconia. Post-hoc comparisons showed that the SBS was significantly higher after 1 week of storage compared to immediately after treatment (P < 0.05), suggesting a time-dependent increase in bond strength (Fig 3).

For all eight groups, specimens that exhibited adhesive failure between cement and zirconia showed a mean SBS that was equal to or higher than the overall group means. The mean SBS values (in MPa) and standard deviations for the APA groups, listed in order of increasing storage time, were: 26.1 ± 8.1, 22.1 ± 6.1, 29.5 ± 2.0, and 24.9 ± 2.5. For the KHF₂-etched specimens, the corresponding values were: 28.1 ± 8.7, 25.7 ± 6.1, 33.5 ± 4.7, and 26.1 ± 4.2.

### Failure Mode

**Fig 4:**
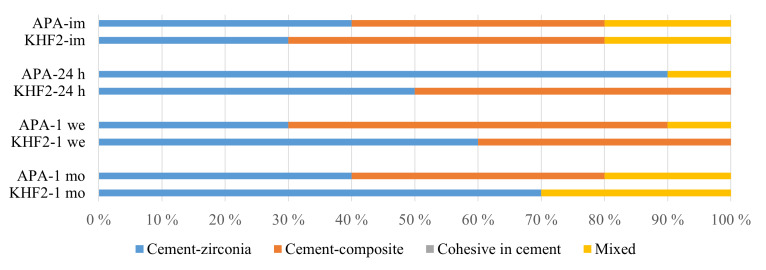
Failure mode after bond strength testing, divided into four groups. Exclusively cohesive failure in cement was not observed. im – immediate; 24 h – 24 hours; 1 we – 1 week; 1 mo – 1 month.

For APA zirconia, the frequency of adhesive failures between cement and zirconia was similar to that between cement and composite in the immediate, 1 week, and 1 month storage groups. However, in the 24 h group, adhesive failure between zirconia and cement predominated and was statistically significantly higher than in the 1 week group (P < 0.05) (Fig. 4).

In the KHF_2_ etched groups, the frequency of adhesive failure between cement and zirconia increased for each storage time, accompanied by a reduction in adhesive failures between cement and composite. Although the increase was not significant (P > 0.05), the frequency of zirconia-cement failures more than doubled between the shortest and longest storage times.

**Fig 5a to h:**
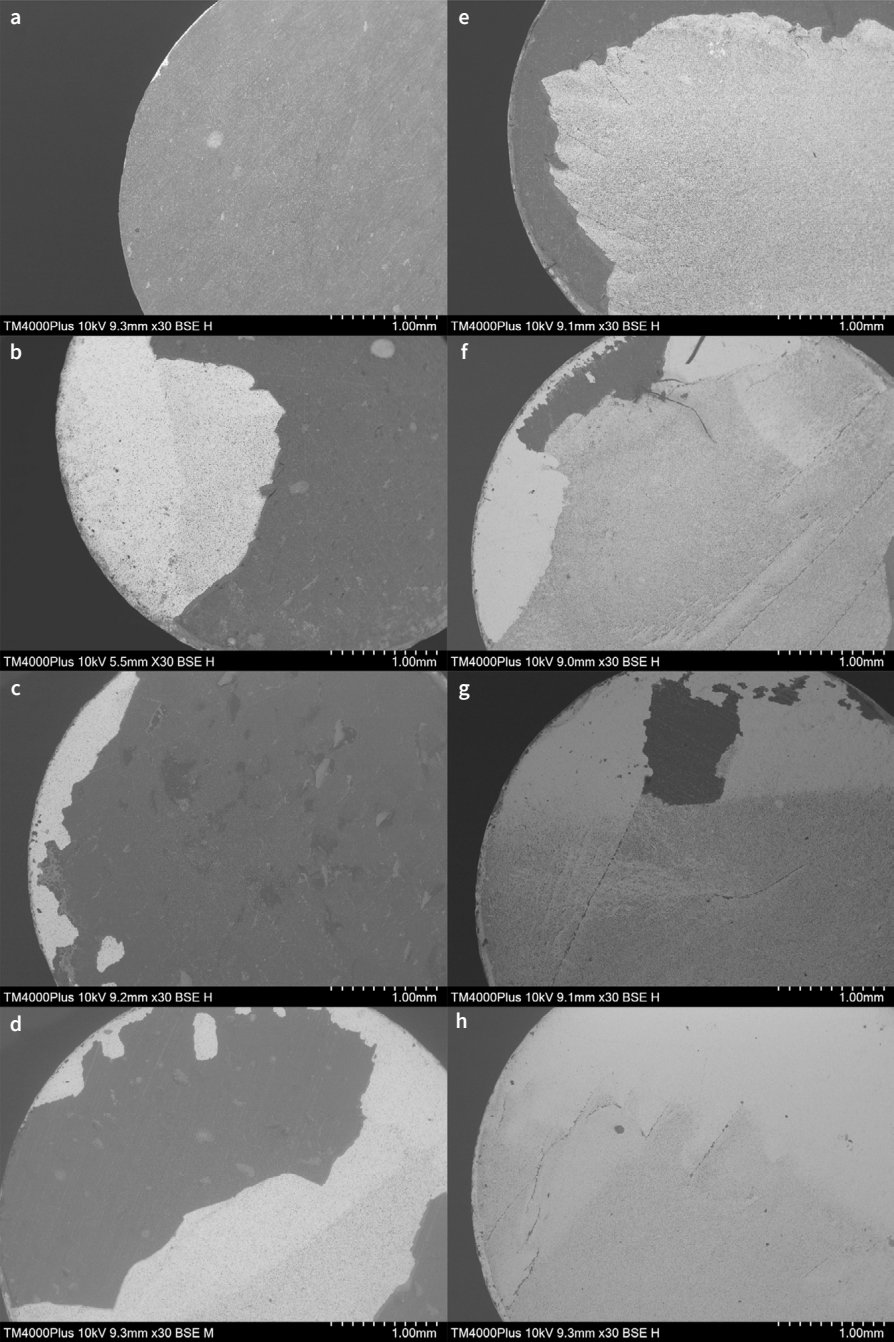
Representative SEM images of zirconia surfaces after bond strength testing: APA zirconia in the immediate, 24 h, 1 week, and 1 month groups, respectively. (a) Adhesive failure between cement and composite. (b) Mixed failure; adhesive between cement and zirconia, cohesive in cement, adhesive between cement and composite. (c) Predominantly adhesive failure between cement and composite, with a small part adhesive between cement and zirconia. (d) Mixed failure; adhesive between cement and zirconia, and cement and composite, cohesive in cement. (e to h) KHF_2_-etched zirconia in the immediate, 24 h, 1 week, and 1 month groups, respectively. (e) Mixed failure; adhesive between cement and composite, cohesive in cement. (f and g) Mixed failure; adhesive between cement and composite, and cement and zirconia, cohesive in dentin. (h) Mixed failure; adhesive between cement and zirconia, cohesive in cement.

A SEM image of selected zirconia cylinders (Fig 5) revealed that cement remnants were retained in surface irregularities created by both APA and KHF_2_ etching, which were not detectable under the light microscope.

## DISCUSSION

The first null hypothesis – that storage time between surface treatment (APA or KHF_2_-etching) of zirconia and cementation has no effect on SFE, SBS or failure mode – must be partially rejected. A significant decrease in SFE from immediate to 1 month was observed for both surface treatments. Although mean bond strength values were not affected by storage time, a higher incidence of adhesive failures between zirconia and composite cement was observed with prolonged storage, particularly for KHF_2_-etched zirconia.^21,28,32^

The second null hypothesis – that surface treatment method (APA or KHF_2_ etching) does not influence SFE, SBS, or failure mode – is partially rejected. KHF_2_-etched zirconia exhibited significantly higher SFE values compared to APA in the immediate, 24 h, and 1 week groups. Despite similar SBS values across all storage times, failure mode analysis revealed a tendency toward more adhesive failures to cement for KHF_2_-etched zirconia after the two longest storage times.

To achieve adequate adhesion between composite cement and the restoration, the cementation surface must have a high wettability to enhance the chemical bonding and facilitate primer and/or composite cement spreading.^29^ Wettability improves with increasing SFE.^34^ A published article by Akazawa et al^1^ reported smaller contact angles between the test liquids and zirconia after KHF_2_ etching compared to APA, demonstrating better wetting of the surface after etching due to higher SFE. The present findings support those results; KHF_2_ etching produced significantly higher SFE values than APA.

For both surface treatments, SFE was highest immediately after the treatment and decreased gradually up to 1 month of storage. This coincides with the result published by Al-Akhali et al,^2^ who observed decreased SFE after up to 72 h storage. In both studies, specimens were stored in closed containers in a dark environment, yet the reduction in SFE is attributed to contamination with hydrocarbons from the surroundings.^2,12^

SFE consists of polar and dispersive components. Polar molecules result in a stronger and more stable attraction, whereas dispersive forces are weaker and unstable due to fluctuating electrons.^16^ A higher proportion of polar components is therefore associated with higher bond strength.^34^ In this study, the polar components decreased significantly over time, from 35% immediately after APA, to 14% after 1 month, and 39% immediately after KHF_2_ etching, to 18% after 1 month. Although a large reduction was observed, the bond strength was not affected. However, when evaluating the failure mode in the light of polar components, an increased frequency of adhesive failures between zirconia and composite cement, especially for KHF_2_-etched specimens, corresponds with the decline in polar components, indicating a weakening of the adhesion to zirconia.

Analysis of SFE should be performed using at least two liquids with known surface tension, ie, deionized water and diiodomethane, in small droplets on the test surface.^15^ Preferably, droplets of each liquid should be applied onto the same specimen. In the present study, only one droplet of liquid could fit the cylinder surface. Therefore, in each of the eight test groups, five cylinders were applied with deionized water, and five were applied with diiodomethane. Thereafter, cylinders were randomly paired to calculate the mean SFE. This randomization might have affected the SFE and distribution of polar and dispersive components.

Akazawa et al^1^ reported significantly higher SBS for KHF_2_-etched zirconia compared to APA when excluding adhesive monomers in primer and cement. The authors attributed their results to increased wettability in combination with a more favorable surface roughness (Ra) after etching compared to APA. Different morphologies after the two treatments have also been reported in other published studies, with the surface after KHF_2_ etching resembling HF etching of glass ceramics.^29,30^ In contrast to the published results by Akazawa et al, the present study detected no significant difference in bond strength between KHF_2_ etching and APA of zirconia after any of the storage times. Application of an MDP containing primer promotes chemical interactions between the restoration and cement^37^ but also deeper penetration into surface irregularities than the cement due to a lower viscosity.^6,34^ The use of an MDP primer after surface treatment when cementing zirconia has been thoroughly studied and is regarded as the best procedure to increase the bond strength of composite cement.^3,8,34,36,37^

In contrast to the study by Akazawa et al, the cemented specimens in the present study were subjected to thermocycling prior to bond strength testing. Thermocycling is a widely accepted *in vitro* aging method for dental materials, designed to simulate the temperature and humidity fluctuation in the oral cavity.^24^ The method, however, represents an accelerated aging process that imposes stress at the material interface due to differences in thermal expansion,^24^ and it is therefore likely to have influenced the SBS values in the present study.

Two-way ANOVA showed a significant effect of storage time on SBS, which the post-hoc test detected was due to a difference between the immediate and 1 week storage groups of KHF_2_ zirconia. Although direct evidence for time-dependent hydrolysis of KHF_2_-derived fluoride residues on zirconia is limited, it is conceivable that partial hydrolysis during short-term storage may generate -OH groups in the surface,^29^ potentially enhancing the bond to phosphate monomers such as 10-MDP.^14^ This mechanism could provide a tentative explanation for the increase in bond strength observed after 1 week in the present study.

When looking exclusively at the specimens with adhesive failure between cement and zirconia, the bond strength was generally higher than or equal to the group means. These results suggest that factors other than the bond between cement and zirconia were important for some of the failures, ie, cement and composite-related factors.^28^ Hence, when failure occurred at other interfaces, it indicated that the zirconia-cement bond withstood the load and the true SBS value for the zirconia bond could have been higher. In a published study by Le et al,^21^ zirconia cemented to zirconia was used for bond strength testing, mitigating the effect of two different substrate surfaces. However, other factors might affect the failure mode with this method, ie, the geometrical configuration of the specimens.^32^

In the KHF_2_-etched group, longer storage appeared to shift the weakest link from the cement-composite interface to the cement-zirconia interface, as suggested by an increased number of adhesive failures after bond strength testing. This coincides with the decreasing SFE values and polar component values after longer storage, suggesting that cementation should be conducted shortly after surface treatment.

Failure mode was assessed under light microscopy due to the macro size of the cylinders. Some specimens were randomly selected for SEM control. In SEM, the surface structure can be studied on a micro or even nano level, but it is a more costly and time-consuming process,^32^ and therefore often only used on selected specimens. SEM revealed a larger amount of cement remnants in surface irregularities than what could be detected under the light microscope, indicating that the frequency of cohesive failures in cement could be higher than reported (Fig 5). Despite the lack of control specimens, roughening of the surface before cementation is important for adhesion to zirconia.^34^ The potential tetragonal to monoclinic crystal phase transformation after APA of 3 mol% yttria zirconia has been the topic of several published studies,^4^ some indicating that the procedure has the potential to strengthen rather than weaken the material by inducing a crystal change from tetragonal to monoclinic.^4^ However, today translucent zirconia holds a significant market share. These materials contain a larger mol% of yttria (up to 8 mol%) that partially or fully stabilizes the crystals in a cubic structure. This crystal structure will not transform and toughen the material when exposed to stresses, resulting in lower mechanical properties.^5,40^ APA of translucent zirconia may rather create defects in the surface that further reduce the mechanical properties.^39^ An advantage of KHF_2_ etching of zirconia compared to APA, is considered to be less structural change of the crystals, thereby lowering the potential for material damage.^29^ Also, the micro-porosities created after etching of zirconia results in a more homogenous surface compared to APA, which further promotes a uniform cement layer thickness and more reliable bond.^10^

Several limitations of the present study should be acknowledged. *In vitro* aging of the specimens was performed using 5,000 thermocycles between 5°C and 55 °C. Because SBS values were similar across surface treatment groups, longer or more extensive thermocycling may influence the results and should be considered in future investigations.^24^ Additionally, the randomized pairing of specimens for SFE measurements introduces some uncertainty. For future studies, specimen surfaces should be large enough to accommodate droplets of both test liquids on the same sample, which would improve the accuracy of SFE determination.

### Acknowledgments

The authors acknowledge the mechanical engineer at NIOM, Dimitri Alkarra, for his assistance with the laboratory work, and the statistician at NIOM, Teferi Mekonnen Yitayew, for his statistical assistance.

The authors declare no conflict of interest.

#### Clinical relevance

Within the limitations of this *in vitro* study, the time elapsed after surface treatment using APA or KHF_2_ etching may influence zirconia surface free energy and, potentially, bond strength and failure mode. No significant differences in bond strength were observed in the present study, but it is conceivable that longer-term aging could lead to a greater decline, which warrants further investigation.

#### Funding

This research did not receive any specific grant from funding agencies in the public, commercial, or not-for-profit sectors.
